# Prenatal Ultrasound Screening for Fetal Anomalies and Outcomes in High-Risk Pregnancies due to Maternal HIV Infection: A Retrospective Study

**DOI:** 10.1155/2013/208482

**Published:** 2013-09-26

**Authors:** A. Reitter, A. U. Stücker, H. Buxmann, E. Herrmann, A. E. Haberl, R. Schlößer, F. Louwen

**Affiliations:** ^1^Department of Obstetrics and Gynecology, University Hospital Frankfurt, Goethe-University, Theodor Stern Kai 7, 60590 Frankfurt, Germany; ^2^Divison of Neonatology, Department of Pediatrics, University Hospital Frankfurt, Goethe-University, Theodor Stern Kai 7, 60590 Frankfurt, Germany; ^3^Department of Biostatistics and Mathematic Modelling, Goethe-University Frankfurt, Theodor Stern Kai 7, 60590 Frankfurt, Germany; ^4^Department of Infectious Diseases, University Hospital Frankfurt, Goethe-University, Theodor Stern Kai 7, 60590 Frankfurt, Germany

## Abstract

*Objective*. To assess the prevalence of prenatal screening and of adverse outcome in high-risk pregnancies due to maternal HIV infection. *Study Design*. The prevalence of prenatal screening in 330 pregnancies of HIV-positive women attending the department for prenatal screening and/or during labour between January 1, 2002 and December 31, 2012, was recorded. Screening results were compared with the postnatal outcome and maternal morbidity, and mother-to-child transmission (MTCT) was evaluated. *Results*. One hundred of 330 women (30.5%) had an early anomaly scan, 252 (74.5%) had a detailed scan at 20–22 weeks, 18 (5.5%) had a detailed scan prior to birth, and three (0.9%) had an amniocentesis. In seven cases (2.12%), a fetal anomaly was detected prenatally and confirmed postnatally, while in eight (2.42%) an anomaly was only detected postnatally, even though a prenatal scan was performed. There were no anomalies in the unscreened group. MTCT occurred in three cases (0.9%) and seven fetal and neonatal deaths (2.1%) were reported. *Conclusion*. The overall prevalence of prenatal ultrasound screening in our cohort is 74.5%, but often the opportunity for prenatal ultrasonography in the first trimester is missed. In general, the aim should be to offer prenatal ultrasonography in the first trimester in all pregnancies. This allows early reassurance or if fetal disease is suspected, further steps can be taken.

## 1. Introduction

The majority of women living with HIV are in their reproductive years (ages 15–49) [1, 2]. The dramatic decrease in the risk of mother-to-child HIV transmission (MTCT) is leading to normality in the lives of couples affected by HIV, who want own children. In Europe, the reduction in MTCT to less than 1% is mainly due to highly active antiretroviral therapy (HAART). Effective HAART is resulting in suppressed viral load (VL); thus, a vaginal birth can be as safe as a planned caesarean section [3, 4]. Avoidance of breastfeeding and postnatal neonatal postexposure prophylaxis (PEP) further supports the effective reduction in MTCT [3–5]. Still there is a fear of higher pregnancy complications in women living with HIV [6]. The literature suggests that there is no increased rate of fetal malformations due to the HIV infection or HAART [6, 7]. A pregnant woman with HIV infection usually has intensified prenatal care including referral for prenatal ultrasound screening [8].

Prenatal ultrasound screening is being offered earlier and earlier [9]. Large studies of noninvasive prenatal screening have already indicated that it will lead to a decrease of invasive prenatal screening procedures such as amniocentesis (AC) or chorionic villi biopsy (CVS) [10]. If invasive prenatal testing is necessary, it can be done, but in these circumstances, HAART should be started prior to the procedure to suppress the VL below the limit of detection. In these cases current, evidence suggests that MTCT is very unlikely; however, studies reporting on the risk of MTCT in invasive prenatal testing are limited due to small study size [11].

HAART is given during pregnancy for two reasons, first to women with an own indication for HAART (they require treatment for their own health) and secondly to pregnant women starting therapy purely as a prophylactic treatment to reduce MTCT. 

The aim of our study was to investigate if pregnant HIV-positive women get referred for special prenatal ultrasound screening services in our tertiary referral center, but also if and at what point the prenatal ultrasonography is performed. Pregnant HIV-positive women usually have a combined antenatal care in a tertiary referral center and with their own gynaecologists. 

As well as the prevalence of prenatal ultrasound screening, prenatal, and postnatal finding was recorded. We hypothesized that the fetal anomaly rate in women with HIV-infection is as low as in all other pregnancies (3–5%) [12, 13]. 

## 2. Materials and Method

HIV-positive pregnant women who presented in our tertiary referral center between January 1, 2002 and December 31, 2012 were included in this retrospective cohort study. 

Only pregnancies ≥24 weeks of gestation were included. Three categories were used: very preterm delivery (24 + 0 to 33 + 6 weeks of gestation), preterm delivery (34 + 0 to 36 + 6 weeks of gestation), and term delivery (≥37 weeks of gestation). 

All data regarding early prenatal screening (as, e.g., nuchal translucency measurements) and fetal anomaly scan at 20 weeks of gestation or at first presentation in our center were recorded. Only scans which were performed in our center were included, reflecting the fact that HIV-positive pregnant women are high risk pregnancies, and high-risk pregnancies are referred to a tertiary center or an equivalent specialized center for prenatal screening [14–17]. An early anomaly scan was defined as a first trimester scan; in the study period, the fetal nuchal thickness was assessed; a formal nuchal translucency measurement was included if measured by appropriately qualified sonographers. A fetal anomaly scan was defined as a detailed scan in the second trimester (usually between 20 to 22 weeks of gestation). All the scans performed at a later gestation in our department prior to birth are recorded separately as late scans in the third trimester. We collected the abnormal prenatal sonographic findings and compared prenatal with postnatal detected malformations.

Malformations were any fetal/neonatal disease, which required either surgery or special pediatric care including chromosomal anomalies [18]. All cases with an AC, MTCT, and any intrauterine or postnatal death were evaluated.

Maternal information included age, ethnicity, gestational age at delivery, gravidity and parity, HAART already before the pregnancy, VL (copies/mL), CD4 count (cells/*μ*L) prior to birth, and other risk factors such as coinfection with HCV. The last recorded VL prior to the delivery was used and classified in three risk groups. In the study, a VL below 50 is considered as negative/undetectable. The last CD4 count prior to birth was noted, and again three categories were used. The mode of delivery was classified as (1) planned caesarean section; (2) in cases of rupture of membranes and/or contraction it was recorded as elective caesarean section in labour; (3) emergency caesarean section; (4) caesarean section after planned vaginal birth; (5) vaginal birth; (6) unplanned vaginal birth and (7) instrumental vaginal delivery. In the unit the first planned vaginal birth was recorded in 2009. Before that time, women were offered elective caesarean section at around 37 + 0 weeks of gestation [3, 8, 19]. With evidence for the safety of the vaginal birth with undetectable VL, the policy in the unit shifted towards planned vaginal birth, and if caesarean section was offered in these cases, the delivery was delayed until >37 weeks of gestation according to the German-Austrian Guidelines [3, 4, 8]. The following neonatal data were included: APGAR score, arterial cord pH (apH), cord base excess (BE) and neonatal weight (stratified according to 10th, 10–90th and >90th percentile). A weight below the 10th percentile was considered to be intrauterine growth retardation (IUGR). 

Information about scan findings was obtained from the record of the ultrasound department, and further information was collected from maternal case notes, pediatric notes, and discharge letters. 

Ethics approval for the retrospective study was obtained from the Ethics Committee at the J. W. Goethe University, Frankfurt (number 30/13).

For categorical variables and nominal variables, frequency tables were used for descriptive statistical analysis. For ordinal and quantitative data, mean and standard deviation (SD) or percentiles were used. These data were further analyzed using the Wilcoxon-Mann-Whitney Test, Kruskal-Wallis Test, Spearman-Correlation, Chi^2^-Test, and Fisher's Exact Test as appropriate. All tests were 2-sided and a *P* value below 0.05 was considered statistically significant. In addition, multivariate logistic regression analysis was performed to identify factors associated with a woman having an early anomaly scan. 

Statistical analysis was performed using IBM SPSS 20 statistics software.

## 3. Results

Overall 330 pregnancies were recorded, with 322 singleton pregnancies (97.6%) and in eight twin gestations (2.4%). One twin pregnancy was conceived due to IVF with first diagnosis of the HIV-infection in the early second trimester. There were 122 preterm deliveries (36.5%) and 90 (26.9%) of these were between 34 and 36 + 6 weeks of gestation. Maternal and neonatal characteristics are presented in Table 1, stratified by pregnancy duration in Table 2. 

The mean age at presentation was 31.1 ± 5.7 years. Nearly half of all the women (49.7%) were primiparae. Two thirds of women (66.4%) were of African ethnicity. In one quarter of women, the diagnosis HIV of infection occurred in the pregnancy. More than three quarters 257 (77.4%) of the births were elective caesarean section. In 29 cases (8.7%), women delivered vaginally. The CD4 count (cells/*μ*L) prior to birth was in the majority of 175 (62.5%) ≥350, in 76 (27%) between 200 and 349, and in 30 (10.7%) <200. The VL (copies/mL) in most women 168 (55.8%) was suppressed below 50 copies in 88 (29.2%) 50–399 and in 45 (15%) ≥400. One hundred and eight women (37.4%) were on no HAART treatment at the beginning of the pregnancy. In 25 (8.9%), a positive anti-HCV test was recorded. The average weight of the newborn was 2837 g (±656). Thirty newborns (9%) were classified as below the 10th percentile [20].

**Figure 1:**
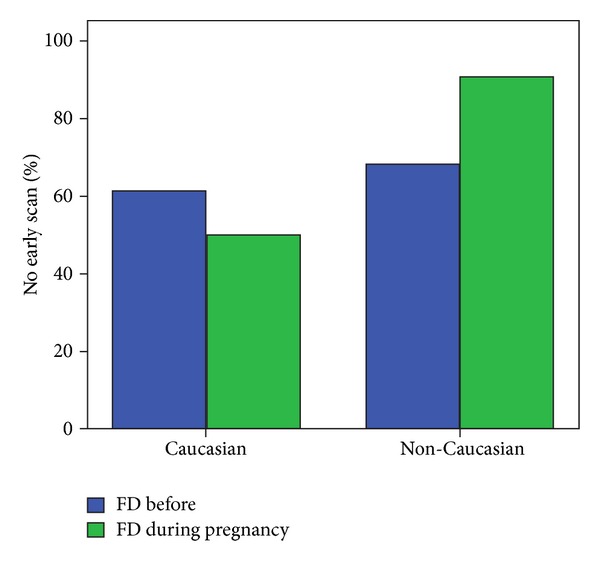
Percentage of women, who had no early scan and factors involved (FD = first diagnosis).

In 100 of the 330 pregnancies (30.5%), we did an early ultrasound assessment. The nuchal translucency was measured in 67 (20.3%) of the 330 cases (NT median 1.22 mm (range 0.6–3 mm)). A multivariate analysis for factors influencing a woman having an early anomaly scan (Table 3) showed that African ethnicity and first diagnosis of HIV during the ongoing pregnancy were factors which significantly could be related to not having early prenatal ultrasound screening (Figure 1). 

Invasive testing (AC) was done in three (0.9%) of 330 cases. Only one case was done at 25 weeks in our department, and we started HAART and performed the AC after VL was fully suppressed. The Karyotype was normal. Two cases were done for advanced maternal age without control of VL and without specific precautions for example, HAART, and both revealed a normal Karyotype. In all of three cases, no MTCT occurred.

In the second trimester in 252 (74.5%) of 330, a detailed anomaly scan at 20–22 weeks was done. In 18 (5.5%) patients, the scan was performed in the third trimester due to late presentation in our unit. In Table 4, fetal and neonatal malformations as well as chromosomal anomalies are presented. In seven cases of 330 cases (2.1%), we diagnosed a fetal malformation. There was no apparent coincidence with HAART or any other recurrence of fetal malformation. Postnatally, all of the seven cases were confirmed, and eight further malformations and two cases with trisomy 21 were detected. The chromosomal anomalies were not suspected. Both women, 33 and 39 years of age, had no early scan or biochemical screening but a scan in our unit (late in the second trimester with no anomalies seen). There were three cases with a skin tag, one nevus sebaceous of the occiput, and one case with a socalled sucking blister on the hand, all considered to be minor. Each of these cases had at least one scan in our department prior to the birth. However, the sucking blister and the nevus were leading to an upgrade in neonatal PEP due to breaking down of protective skin barrier, and one newborn presented with a small omphalocele which was not seen prior to birth. All of these babies were born by elective caesarean section. The overall fetal malformation rate (including the minor anomalies) was 4.5% [18]. 

In Table 5, the fetal and postnatal mortalities are recorded. In our cohort, we had six cases of intrauterine or postnatal loss and all were born by caesarean section. We present in Table 6 the three cases of MTCT. All of the three newborns were delivered by caesarean section, and all were preterm (33 + 6, 36 + 3 and 36 + 4 weeks of gestation). In all cases, the VL was detectable, all women were on HAART, and one woman was coinfected with HCV. One woman had already a vertically infected child, and she had a poor compliance. 

## 4. Conclusion 

There are conflicting results regarding the risks for HIV-positive mothers for possible adverse effects in their pregnancies [5, 6]. In our study, we confirm the low fetal malformation rate of 4.5% in women living with HIV. There are different national registers collecting data on HAART and pregnancy outcome (e.g., APR: Antiviral Pregnancy Registry; NSHPC: National Study of HIV in Pregnancy and Childhood (UK); ECS: European Collaborative Cohort; EPF—French Perinatal Cohort) [4, 21–24]. These registers confirm the same malformation rate in women taking HAART as in the general population (3–5%) [12, 13].

Prenatal screening was found to be successful in diagnosing major fetal malformations. The postnatal anomalies were minor ones (skin tag, sucking blister) or missed due to minimal extend (omphalocele). The two cases with postnatal trisomy 21 were missed prenatally but were not seen in typical screening periods. There were no anomalies in the unscreened population.

A change in treatment policies is evident over the 11 years of the study, reflected in the changes in delivery mode over time and the gestational age at delivery [8]. A high preterm delivery rate is confirmed by other groups [6]. In our population, 26.9% are late preterm deliveries (34–36 + 6 weeks of gestation) and are mostly iatrogenic due to early caesarean section as in other studies and in the past [25]. The updated national German-Austrian Guidelines now delay caesarean section to term in women with suppressed VL [8]. The numbers of women with fully suppressed VL (VL < 50 copies/mL) (*P* < 0.001) and CD4 cells ≥350 (*P* > 0.20) prior to birth increased over the last years. 

There are two important screening intervals during the prenatal period. The first is early screening which should take place between 11 + 0 and 14 + 0 weeks of gestation [15]. This early screening was introduced by Nicolaidis in 1992 as a combined method of screening (including ultrasound screening and two maternal biochemical markers: free human chorionic gonadotropin (free hCG*β*) and pregnancy-associated plasma protein A (PAPP-A) [26–28]. In Germany this test is not covered by the national health system and there for is paid by the woman herself. Usually at that time an early anomaly ultrasound scan can be performed, which is covered by the health system. The second screening interval is the anomaly scan at 20–22 weeks of gestation [17, 29]. 

The prevalence of first trimester screening of 97.5% in a low risk general population has been demonstrated [30]. 

We demonstrate that prenatal screening is offered and available, but that the early screening interval is missed, as only 30.5% women get referred for early anomaly scan. Even so some women may have chosen not to undergo testing for ethical and cultural reasons. As a limitation of our data it could be that the screening which is done at the community-based care is missed, but as indicated usually, it warrants a referral to a highly qualified and specially trained team [16, 29].

In our study, population the majority of 188 (66.4%) women were of African origin, and in 79 (24.2%), the diagnosis HIV-infection occurred in the pregnancy, both factors were significantly related to having no early prenatal screening. Tariq et al. are reporting on late booking for antenatal care in non-Caucasian women compared with caucasian women regardless of time of diagnosis of HIV-infection [31].

Three cases of MTCT are low (0.9%) and confirmed by other groups (reporting MTCT rates of 0.1%–1.3%) [3, 4]. However, looking back in our data, the viral control has improved dramatically over the last 11 years; in 55.8% of all pregnancies, the VL is <50. 

National health systems vary, and a complete first trimester screening (with inclusion of biochemical serum markers) has not been established on a national basis for high risk pregnancies in some European countries. In our cohort, two cases of trisomy 21 occurred, and the question remains open if these two cases could have been traced in a complete first trimester screening. In one pregnancy, an AC was performed due to suspected chromosomal anomaly, which revealed a normal karyotype. It was done after starting HAART and just after the VL was fully suppressed. Due to the time required to initiate HAART and to have a suppressed VL in HIV-positive pregnancies, invasive testing will be very likely to happen in the second trimester which will then raise the difficult ethical questions about late termination of pregnancy when an abnormal result is obtained [32, 33]. Data in HIV-positive pregnancies reporting on AC is available [32–34]. First trimester screening (including maternal markers as free hCG*β* and PAPP-A) has been investigated in pregnant women living with HIV. Some groups feel that maternal markers could be less reliable than those in HIV-negative women [34, 35]. In our study data from 2002 to 2012, in the first years, the nuchal translucency was assessed but not formally measured. This could be due to the delay in having certified specially trained sonographers involved. 

In the future, the new methods of chromosome-selective sequencing of maternal plasma cell-free fetal DNA (cfDNA) in noninvasive prenatal testing (NIPT) are valuable especially for our study group due to no risk of MTCT [27]. At present this interesting method is not widely available, and more data of this new method are needed. 

## Figures and Tables

**Table 1:** Maternal and neonatal characteristics.

Characteristics	Value (total pregnancies *n* = 330, total newborns *n* = 338)
Maternal age at delivery (±SD)	31.05 ± 5.7

Gravidity	*N* = 326
1	89 (27.3%)
2	104 (31.9%)
3	71 (21.8%)
4	38 (11.7%)
≥5	24 (7.2%)

Parity	*N* = 326
1	162 (49.7%)
2	108 (33.1%)
3	36 (11%)
4	12 (3.7%)
≥5	8 (2.4%)

Duration of pregnancy (weeks of gestation)	*N* = 334
24 + 0–33 + 6	32 (9.6%)
34 + 0–36 + 6	90 (26.9%)
>37 + 0	212 (63.5%)

Ethnicity	*N* = 283
Caucasian	95 (33.6%)
African	188 (66.4%)

HIV diagnosis during pregnancy	*N* = 327
Yes	79 (24.2%)
No	243 (75.8%)

Mode of delivery	*N* = 332
Planned caesarean section	257 (77.4%)
Caesarean section in labor	30 (9%)
Emergency caesarean section	1 (0.3%)
Caesarean section after trial of vaginal birth	13 (3.9%)
Spontaneous vaginal delivery	29 (8.7%)
Unplanned vaginal delivery	1 (0.3%)
Operative vag delivery (e.g., forceps)	1 (0.3%)

CD4 count at delivery (cells/*μ*L)	*N* = 281
<200	30 (10.5%)
200–349	76 (27%)
≥350	175 (62.5%)

Viral load at delivery (copies/mL)	*N* = 301
<50	168 (55.8%)
50–399	88 (29.2%)
≥400	45 (15%)

HAART before the beginning of pregnancy	*N* = 289
Yes	181 (62.6%)
No	108 (37.4%)

HCV (positive anti-HCV test)	*N* = 280
Yes	25 (8.9%)
Negative	255 (91.1%)

5-min APGAR	*N* = 330
<4	0
<7	5 (1.5%)
7–10	325 (98.5%)

Arterial cord pH	*N* = 327
<7.0	0
7.0-<7.1	2 (0.6%)
7.1-<7.2	16 (4.9%)
≥7.2	309 (94.5%)

Base excess (±SD)	*N* = 323 −2.98 ± 2.15

Weight (g) (±SD)	*N* = 333 2837 ± 656

Percentile	*N* = 335
<10th	30 (9%)
10–90	291 (86.8%)
>90th	14 (4.2%)

Early anomaly scan (11 + 0–14 + 0 weeks of gestation)	*N* = 100 30.5%

Nuchal translucency	*N* = 67 20.3%

Anomaly/detailed scan 20–22 weeks of gestation	*N* = 252 74.5%

Anomaly scan any time later in pregnancy	*N* = 18 5.5%

Fetal/neonatal anomalies in total	*N* = 15 4.5%

MTCT in total	*N* = 3 0.9%

MTCT: mother-to-child transmission; SD: standard deviation.

**Table 2:** Maternal and neonatal characteristics according to duration of pregnancy.

	Duration of pregnancy in weeks			*P* value^1^
	24 + 0–33 + 6 *N* = 32	34 + 0–36 + 6 *N* = 90	37 + 0–42 *N* = 212	
Maternal age at delivery (±SD)	32.5 ± 5.8	30.2 ± 5.8	31.2 ± 5.6	*P* = 0.102

Gravidity *n* = 326				*P* > 0.20
1	7 (24.1%)	21 (24.7%)	68 (28.8%)	
2	9 (31%)	28 (32.9%)	67 (31.6%)	
3	7 (24.1%)	19 (22.4%)	45 (21.2%)	
4	3 (10.3%)	9 (10.6%)	26 (12.3%)	
>5	3 (10.3%)	8 (10.1%)	13 (10.7%)	

Parity *n* = 326				*P* > 0.20
1	16 (55.2%)	42 (49.4%)	104 (49.1%)	
2	6 (20.7%)	30 (35.3%)	72 (34%)	
3	4 (13.8%)	9 (10.6%)	23 (10.8%)	
4	0	3 (3.5%)	9 (4.2%)	
>5	3 (10.3%)	1 (1.2%)	4 (1.8%)	

Ethnicity *n* = 283				*P* > 0.20
Caucasian	5 (22.7%)	27 (38.6%)	63 (33%)	
African	17 (77.3%)	43 (61.4%)	128 (67%)	

HIV diagnosis during pregnancy *n* = 327				*P* > 0.20
Yes	9 (30%)	19 (22.1%)	51 (24.2%)	
No	21 (70%)	67 (77.9%)	160 (75.88%)	

Mode of delivery *n* = 332				*P* < 0.001
Planned caesarean section	26 (83.87%)	70 (78.6%)	161 (75.94%)	
Caesarean section during labor	5 (16.13%)	15 (16.85%)	10 (4.72%)	
Emergency caesarean section	0	0	1 (0.47%)	
Caesarean section after trial of vag birth	0	0	13 (6.13%)	
Spontaneous vaginal delivery	0	3 (3.37%)	26 (12.26%)	
Unplanned vaginal delivery	0	1(1.12%)	0	
Instrumental vaginal delivery (e.g., Forceps)	0	0	1 (0.47%)	

CD4 count at delivery (cells/*μ*L)*n* = 281				*P* = 0.075
<200	2 (8%)	6 (8.3%)	22 (12%)	
200–349	12 (48%)	14 (19.4%)	50 (27.2%)	
>350	11 (44%)	52 (72.2%)	112 (60.9%)	

Viral load at delivery (copies/mL) *n* = 301				*P* < 0.001
<50	9 (36%)	27 (34.6%)	132 (66.7%)	
50–399	7 (28%)	32 (41%)	49 (24.7%)	
≥400	9 (36%)	19 (24.4%)	17 (8.6%)	

HAART before beginning of pregnancy *n* = 289				*P* > 0.20
Yes	17 (66.7%)	41 (55.4%)	123 (65.4%)	
No	10 (33.3%)	33 (44.6%)	65 (34.6%)	

HCV (positive anti-HCV test) *n* = 280				*P* > 0.20
Yes	2 (8%)	10 (13.2%)	13 (7.3%)	
Negative	23 (92%)	66 (86.8%)	166 (92.7%)	

5-min APGAR *n* = 330				
<4	0	0	0	*P* < 0.001
<7	5 (17.24%)	0	0	
7–10	24 (82.75%)	90 (100%)	211 (100%)	

Arterial cord blood levels (*n* = 327)				*P* = 0.088
<7.0	0	0	0	
7.0-<7.1	0	0	2	
7.1-<7.2	3 (10%)	3 (3.4%)	10 (4.83%)	
>7.2	27 (90%)	85 (96.6%)	197 (95.17%)	

Base excess (*n* = 323) (±SD)	*N* = 28 −3.77 ± 2.49	*N* = 87 −2.65 ± 2.09	*N* = 208 −3.02 ± 2.1	*P* = 0.091

Fetal weight (g) (*n* = 333) (±SD)	*N* = 32 1573.28 ± 517.98	*N* = 90 2634.56 ± 463.12	*N* = 211 3115.14 ± 460.06	*P* < 0.001

Percentile (*n* = 335)	*N* = 32	*N* = 90	*N* = 211	*P* > 0.20
<10	1 (3.3%)	7 (8.2%)	21 (9.9%)	
10–90	28 (84.7%)	80 (88.3%)	183 (86.7%)	
>90	3 (10%)	3 (3.5%)	7 (3.3%)	

Early anomaly scan (11–14 weeks of gestation) *n* = 100	11 (36.7%)	24 (28.2%)	65 (30.7%)	*P* > 0.20

Anomaly scan (second trimester) *n* = 252	26 (81.2%)	68 (75.6%)	158 (74.5%)	*P* > 0.20

Anomaly scan at later stage *n* = 18 (5.5%)	2	8	8	*P* > 0.20

Prenatally seen anomalies *n* = 7 (2.1%)	3	2	2	*P* > 0.20

Postnatal confirmed anomalies	4	2	9	*P* > 0.20
In total *n* = 15 (4.5%)				

^1^ *P* values were calculated without significance correction. Kruskal-Wallis test was used for maternal age, gravidity and parity, APGAR score, apH, vpH, BE, fetal weight and percentile, fetal length, head circumference, early anomaly scan, anomaly scan. Chi² test was used for the other characteristics. SD: standard deviation.

**Table 3:** Results of multivariate analysis on factors for a woman to have an early anomaly scan.

	OR	95% CI for OR	*P* value
Ethnicity	2.008	1.155–3.491	0.013
First diagnosis in present pregnancy	2.085	1.033–4.209	0.040
Gravity	0.767	0.581–1.013	0.062
Parity	1.134	0.756–1.702	0.543
Birth weight	1.000	0.999–1.001	0.710
Gestation at delivery	0.950	0.797–1.133	0.596

OR: odds ratio; CI: confidence interval.

**Table 4:** Fetal and neonatal malformation/chromosomal anomalies.

HAART	Diagnosis prenatally	Early scan (first trimester)	Detailed scan (second trimester)	Diagnosis postnatally	Invasive testing/Karyotype	Maternal coinfection	Duration of pregnancy in weeks + days	Outcome
CBV TDF T20	CDH	No	Yes	CDH	No	HCV	36 + 4	After operation alive and well

CBV NVP	VSD	Yes	Yes	VSD	No	HCV HBV	33 + 6	MTCT

CBV NVP	Hydrocephalus, radial deviation of hands	No	Yes	confirmed	Yes (normal Karyotype)	No	33 + 1	Died at 1 day

CBV NVP	Dandy Walker malformation	No	Yes	confirmed	Postnatally unbalanced translocation^1^	No	36 + 1	Died with 3 months

TVD AZT	MCDK Potter 2a	No	Yes	confirmed	Yes	No	37 + 1	Alive and well

CBV NVP	Heart defect (ASD and dextrocardia)	No	Yes	confirmed	No	No	37 + 5	Alive and well

TDF NVP 3TC	No	Yes	Yes	Skin tag (manubrium)	No	No	37 + 3	Alive and well

TVD AZT	No	Yes	Yes	Skin tag (finger)	No	No	36 + 1	Alive and well

TVD AZT	No	No	Yes	Skin tag (finger)	No	NO	37 + 714	Alive and well

TVD NVP	No	No	Yes	ASD	No	No	37 + 1	Alive and well

TZV TDF RAL RTV DRV	No	Yes	Yes	Oesophageal atresia	No	Osophageal correction postnatally	33	Alive and well

AZT TVD LPV	No	No	Yes	VSD	No	No	41 + 3	Alive and Well

AZT TVD SQV RTV	No	No	Yes	Trisomy 21	Yes, postnatally	No	38 + 0	Alive and well

LPV/r 3TC TDF AZT	No	No	Yes	Trisomy 21 VSD and small ASD	Yes, postnatally	No	32 + 4	Alive and well

TVD LPV/r	No	Yes	Yes	Omphalocele	No	No	38 + 3	Alive and well

TVD LPV/r	No	Yes	Yes	Sucking blister	No	No	37 + 4	Alive and well

NVP TVD	No	Yes	Yes	Nevus sebaceous	No	No	37 + 2	Alive and well

^1^46,xy,der(5)t(3;5)(p25.1p15.31).

46,xy,der(5)(5pter>5p15.31:3p25.1>3pter).

CDH: congenital diaphragmatic hernia; ASD: atrial septal defect; VSD: ventricular septal defect; MCDK: multicystic dysplastic kidney disease (Potter II); CBV: zidovudin/lamivudin; TDF: tenofovir; T20: enfuvirtide; NVP: nevirapin; AZT: zidovudin; 3TC: lamivudin; RAL: raltegravir; DRV: darunavir; SQV: saquinavir; RTV: ritonavir; TVD: tenofovir/emtricitabin; LPV/r: lopinavir/ritonavir.

**Table 5:** Neonatal Mortality.

Year	Mode of delivery	Gestational age at delivery	Death	HAART	Fetal/neonatal disease
2003	Planned caesarean section	33 + 1	First day of life	CBV NVP EFV in First trimester	Complex fetal anomaly (hydrocephalus, radial deviation)

2003	Planned caesarean section	36 + 1	3 months	CBV NVP	Dandy Walker malformation Chromosomal anomaly^1^

2007	Planned caesarean section (twins)	32 + 2	9 months	AZT TVD	Sudden infant death

2008	Laparotomy (uterine rupture) and hysterectomy due to placenta percreta	37 + 6	Intrauterine death	AZT RTV SQV 3TC	Intrauterine death

2008	Laparotomy and caesarean section	25 + 2	1 day	KVX NVP	Uterine rupture after fibroidectomy prior to pregnancy

2009	Planned caesarean section	29 + 3	4 weeks	AZT TVD	Volvulus

^1^46,xy,der(5)t(3;5)(p25.1p15.31).

46,xy,der(5)(5pter>5p15.31:3p25.1>3pter).

CBV: zidovudin/lamivudin; NVP: nevirapin; EFV: efavirenz; AZT: zidovudin; TVD: tenofovir/emtricitabin; RTV: ritonavir; SQV: saquinavir; 3TC: lamivudin; KVX: abacavir/lamivudin.

**Table 6:** Mother-to-child transmission.

Year	Mode of delivery	Gestational age at delivery	VL at delivery	Risk of transmission	HAART	Coinfection
2003	Planned caesarean section	33 + 6	90	High	CBV NVP	HCV HBV
2004	Planned caesarean section	36 + 4	1900	Medium	CBV NVP	No
2010	Planned caesarean section	36 + 3	4830	Medium	NVP TVD T20	No

CBV: zidovudine/lamivudin; NVP: nevirapine; TVD, tenofovir/emtricitabin; T20: enfuvirtide.

HCV: hepatitis C; HBV: hepatitis B.
